# The Arg108Cys Variant of Methylmalonyl-CoA Mutase: Clinical Implications for the Mexican Population Based on Molecular Dynamics and Docking

**DOI:** 10.3390/ijms26072887

**Published:** 2025-03-22

**Authors:** Marcela Vela-Amieva, Timoteo Delgado-Maldonado, Enrique Ortega-Valdez, Gildardo Rivera, Gabriel López-Velázquez, Cynthia Fernández-Lainez

**Affiliations:** 1 Laboratorio de Errores Innatos del Metabolismo y Tamiz, Instituto Nacional de Pediatría, Ciudad de México 04530, Mexico; 2 Laboratorio de Biotecnología Farmacéutica, Centro de Biotecnología Genómica, Instituto Politécnico Nacional, Reynosa 88710, Mexico; 3 Laboratorio de Biomoléculas y Salud Infantil, Instituto Nacional de Pediatría, Ciudad de México 04530, Mexico

**Keywords:** methylmalonic acidemia, propionate defects, inborn errors of metabolism, rare diseases, genetic diseases

## Abstract

Methylmalonic acidemia (MMA) is a genetic condition associated with intellectual disability and a high mortality rate. It is caused by pathogenic variants in the *MMUT* gene, which codes methylmalonyl-CoA mutase enzyme (MUT). In the Mexican population, the variant NM_000255.4:c.322C>T or p.(Arg108Cys) is the most frequently found, but its structural pathogenic effect is scarcely studied. To describe the clinical picture of p.(Arg108Cys) homozygous patients and to predict its structural pathogenic effect, we performed an analysis of the medical files from six MMA Mexican p.(Arg108Cys) homozygous patients. The structural changes in MUT caused by this variant were analyzed through molecular dynamics simulations (MDS) and docking and compared with the wild-type (Wt) enzyme. The main clinical symptoms presented by the patients were feeding difficulties, lethargy, and neurodevelopmental delay, with a predominance of early-onset phenotype and a mortality rate of 83%. We found significant structural changes in MUT structure, particularly in the catalytic domain, with increased volume cavity, shortening of the binding substrate tunnel, and aberrant accommodation. Also, the dimerization interface area increased from 1343 Å^2^ in the Wt to 3386 Å^2^, and the dimer formation involved a different set of amino acids. The NM_000255.4:c.322C>T or p.(Arg108Cys) *MMUT* variant is associated with a severe outcome in MMA Mexican patients, and the enzyme was associated with ostentatious topological changes in the secondary and tertiary structure, which impacted the catalytic domain, the accommodation of the substrate, and the dimerization interface. Further ex vivo functional studies are needed to confirm these predictions, such as enzymatic activity measurements in fibroblasts of patients.

## 1. Introduction

Methylmalonyl-CoA mutase (MUT) is an adenosylcobalamin (AdoCbl)-dependent enzyme that catalyzes the isomerization of methylmalonyl-CoA to succinyl-CoA. This enzyme belongs to the class I group of isomerases [1]. MUT participates in the propionate metabolic pathway, which catabolizes propionyl-CoA precursors such as branched-chain amino acids, methionine, threonine, odd-chain fatty acids, and the side chain of cholesterol. MUT catalyzes the last step of the pathway for the obtention of succinyl-CoA, which concomitantly leads to the Krebs cycle for further energy obtention. MUT is expressed mainly in the liver, kidney, and adrenal glands [2].

MUT is a 750-amino-acid protein with a molecular mass of 75 kDa. It is encoded in the *MMUT* gene (OMIM 609058) and acts in the mitochondrial matrix [3]. The functional unit of MUT is a homodimer. Each monomer is composed of a mitochondrial leader sequence (amino acids 1 to 32), followed by the dimerization domain (amino acids 33 to 85), catalytic domain (amino acids 86–423), linker domain (amino acids 424 to 577), and AdoCbl binding domain (amino acids 578 to 750) [4]. The proteins named metabolism of cobalamin-associated A (MMAA) and metabolism of cobalamin-associated B (MMAB) are chaperones that form a protein complex with MUT and assist it during catalysis. They help to load and offload the cofactor AdoCbl. This assistance is needed since, in every catalytic cycle, unpaired and reactive electrons are generated, which make MUT susceptible to oxidative inactivation and lead to the formation of hydroxocobalamin (OH-2 Cbl), which inactivates the enzyme unless the repaired cofactor is exchanged [5].

Mutations in the *MMUT* gene can cause MUT deficiency, leading to methylmalonic acidemia (MMA). Thus, MMA is a genetic condition in which the disruption of the conversion of methylmalonyl-CoA to succinyl-CoA provokes the abnormal accumulation of metabolites such as propionyl carnitine (C3), propionyl glycine, methylcitric acid, and methylmalonic acid, among others. This leads to the blocking of other critical metabolic pathways, such as the urea cycle, and the disruption of anaplerosis of the Krebs cycle with concomitant energy deficiency, as well as defective autophagy and lysosomal dysfunction [6,7,8,9].

MMA patients could present a severe affectation characterized by biochemical abnormalities such as ketoacidosis crises, hyperammonemia, and blood glucose dysregulation. Clinically, patients can present with vomiting, feeding refusal, neurological deterioration with lethargy, hypotony, seizures, failure to thrive, spasticity, hematological data (anemia, neutropenia, thrombocytopenia, or pancytopenia), and pancreatitis; kidney and immunological functions could also be affected [10]. The disease is characterized by acute and recurring metabolic crises, which may result in complications such as multiorgan failure and permanent intellectual disability [11]. Conventional treatment includes the nutritional restriction of propionate precursors (low natural protein diet covering the age-appropriate total protein requirements) complemented with levocarnitine and ammonia scavengers or extracorporeal detoxification. Liver or combined kidney and liver transplantation have also been considered as therapeutic approaches, with variable results [12]. Unfortunately, despite the early diagnosis, until now, the treatment has not been entirely effective, and MMA patients face a poor prognosis with high mortality [13,14].

In Mexico, MMA prevalence is unknown, but among the patients attended in our metabolic reference center, it is the most frequent organic acidemia registered [15]. Our group recently reviewed the genotypic spectrum of *MMUT*, observing that the “Hispanic” NM_000255.4:c.322C>T or p.(Arg108Cys) variant was the most frequently found, and in the homozygous state, it is related to high mortality [16]. This variant has also been associated with a severe, early-onset clinical presentation [17]. However, its structural pathogenic effect remains incompletely understood. This study aims to describe the clinical picture of six patients with methylmalonic acidemia who are homozygous for the NM_000255.4:c.322C>T or p.(Arg108Cys) *MMUT* variant. We also aimed to predict the structural pathogenic effect of this variant using in silico tools to provide structural insights into the protein that supports its clinical severity.

## 2. Results

### 2.1. Clinical and Demographic Characteristics of MUTd Patients

From the six unrelated patients with the homozygous genotype c.[322C>T];[322C>T] or p.[Arg108Cys];[Arg108Cys], three were male, and three were female. Consanguinity was documented only in one family (1/6, 16.66%). Two of the families had a history of neonatal deaths (one and two cases, respectively), with a clinical presentation suggestive of metabolic disease. None of them received a specific diagnosis or genetic counseling. All patients were born full term, with birth weight and height in normal ranges for the gestational age. At birth, one out of the six patients required resuscitation maneuvers, mechanical ventilation, and hospitalization. All the other patients were discharged as healthy newborns. None of the patients were screened for propionate defects, as these diseases are not included in the Mexican mandatory newborn screening.

**Figure 1:**
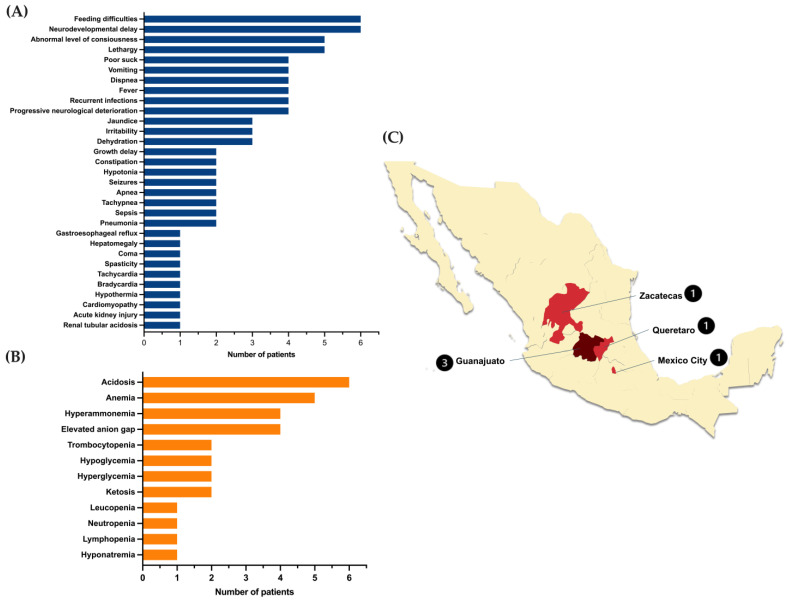
Clinical (**A**) and biochemical characteristics (**B**) of the MUTd patients homozygous for the NM_000255.4:c.322C>T or p.(Arg108Cys) variant and geographical distribution of the families in Mexico. (**C**) The numbers in a circle indicate the cases in each location.

The mean age at symptom onset was 0.5 ± 0.5 months (range 1 day–3.0 months); the mean age at diagnosis was 6.5 ± 1.4 months (2.8–12.3); and the diagnostic odyssey was 9.33 months (3.7–9.3). Since none of the patients were detected by newborn screening, all of them initiated the nutritional treatment lately (more than six months old), all except one patient are deceased (83.3%), and they died at an average age of 49 ± 30 months (6 months–14 years). The registered causes of death were mainly metabolic decompensation complicated with respiratory infections, sepsis, pancreatitis, renal failure, and septic shock. Figure 1 shows the clinical and biochemical characteristics of the studied patients at the moment of diagnosis and the geographic distribution of the families.

### 2.2. Molecular Dynamics of Human Methylmalonyl-CoA Mutase After 100 Ns Simulation

**Figure 2:**
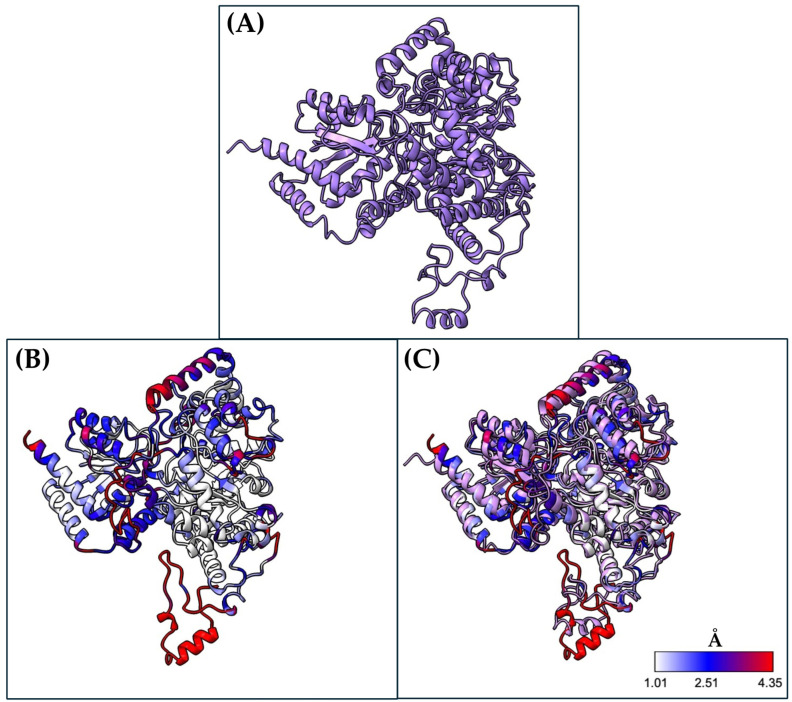
Structures from MDS of human methylmalonyl-CoA mutase after 110 ns simulation. Wt MUT after MDS (**A**) is compared by amino acids RMSD with the NM_000255.4:c.322C>T or p.(Arg108Cys) variant after MD, (**B**) and 3D structural alignment of both structures after MDS are shown. (**C**) RMSD corresponds to the structures extracted from the MD simulations (frames from 105–110 ns). Structures are visualized in ribbons.

Molecular dynamics revealed significant changes in the MUT tertiary structure, particularly in the catalytic domain. We show the monomer of Wt MUT (Figure 2A) and the NM_000255.4:c.322C>T or p.(Arg108Cys) variant (Figure 2B) after MDS. Both structures are aligned based on their 3D conformation, with differences evaluated using the RMSD of the main chain alpha carbons (Figure 2C). A color-coded guide is provided to compare the degree of deviation from the native structural arrangement in the mutant protein.

#### RMSD, RMSF, Rgyr, and SASA Analyses

**Figure 3:**
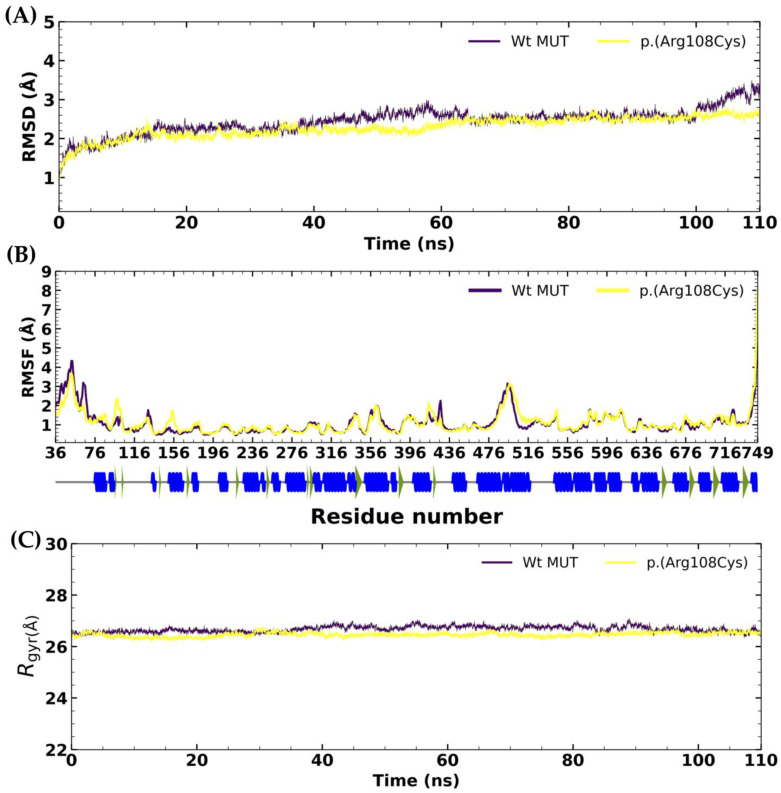
Root mean square deviation (RMSD), (**A**) root mean square fluctuation (RMSF) (**B**), and radius of gyrus (Rgyr) (**C**) plots of the Wt MUT and p.(Arg108Cys) variant of methylmalonyl-CoA mutase. The figures represent the average values of three MDS replicates. For the Wt MUT, the mean RMSD was 2.44 ± 0.32 Å, the mean RMSF was 1.08 ± 0.63 Å, and the mean Rgyr was 22.98 Å ± 0.10. For the p.(Arg108Cys) variant, the mean RMSD was 2.28 ± 0.26 Å, the mean RMSF was 1.10 ± 0.60 Å, and the mean Rgyr was 22.79 Å ± 0.07.

The global root mean square deviation (RMSD) was calculated from the mean of three MDS replicates. The proteins Wt MUT and NM_000255.4:c.322C>T or p.(Arg108Cys) variant showed acceptable stability in terms of RMSD. The RMSD analysis revealed minimal fluctuations throughout the MDS trajectory (Figure 3A). The mean RMSD of the Wt MUT was 2.44 ± 0.32 Å, with a maximum RMSD value of 3.48 Å and a minimum RMSD value of 0.29 Å. In the case of the p.(Arg108Cys) variant, the mean RMSD was 2.28 ± 0.26 Å, while the maximum RMSD value was 2.80 Å, and the minimum was 0.29 Å. These results indicate an adequate stability of these proteins under the evaluated conditions.

Residue RMSD was calculated as the root mean square fluctuation (RMSF), representing the residue-level fluctuations. This permitted us to analyze the local changes through atomic position changes along the protein chain (Figure 3B). During the simulation, the RMSF calculation for the Wt MUT showed fluctuations between 6.52 Å and 0.29 Å, with these values being the maximum and minimum, respectively. The mean RMSF of the Wt MUT was 1.08 ± 0.63 Å. On the other hand, the RMSF calculation for the p.(Arg108Cys) variant during the simulation showed fluctuations between 8.03 Å and 0.51 Å, again identifying these as the maximum and minimum values, respectively. The mean RMSF of the p.(Arg108Cys) variant was 1.10 ± 0.60 Å. The fluctuations during the MDS trajectory for both proteins were below 2.0 Å. One of the most remarkable changes was recorded in the binding pocket region during molecular simulations, where a single-point mutation could create relevant changes in the methylmalonyl-CoA (substrate) environment.

Also, to evaluate both proteins’ compactness, shape, and stability, we calculated the radius of gyration (Rgyr). Rgyr is a measurement of how spread-out atoms are in a molecule and is calculated from the RMSD distance of all atoms from the center of mass of each protein during the MDS. Our results showed that the Rgyr of Wt MUT is lower than the p.(Arg108Cys) (Figure 3C). This indicates a tighter Wt MUT packing structure than p.(Arg108Cys), suggesting increased flexibility and less stability for the latter. The mean Rgyr was 22.98 Å ± 0.10 for Wt MUT, with 23.5 Å and 22.68 Å as maximum and minimum values, respectively. On the other hand, the mean Rgyr was 22.79 Å ± 0.07 for p.(Arg108Cys), with 23.04 Å and 22.57 Å as maximum and minimum values, respectively.

**Figure 4:**
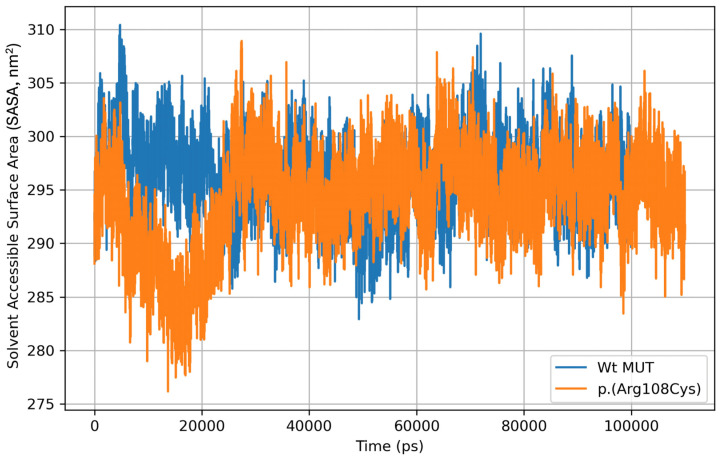
Solvent-accessible surface area (SASA) during the MD simulation of the Wt MUT and p.(Arg108Cys) variant. The graph represents the mean ± standard deviation of SASA for Wt MUT (296.20 ± 3.60 nm^2^) and for the p.(Arg108Cys) variant (294.25 ± 4.54 nm^2^) from three MDS replicates.

Solvent-accessible surface area (SASA) is described as the area of the protein exposed enough to interact with neighboring solvent molecules. The mean SASA from three MDS replicates for Wt MUT was 296.20 nm^2^ ± 3.60, with 310.43 nm^2^ and 282.90 nm^2^ as maximum and minimum values, respectively. On the other hand, the mean SASA for the p.(Arg108Cys) variant was 294.25 nm^2^ ± 4.54, with 308.93 nm^2^ and 276.15 nm^2^ as maximum and minimum values, respectively (Figure 4).

### 2.3. Long-Range Changes Attributable to the Amino Acid Residue Change at Position 108

**Figure 5:**
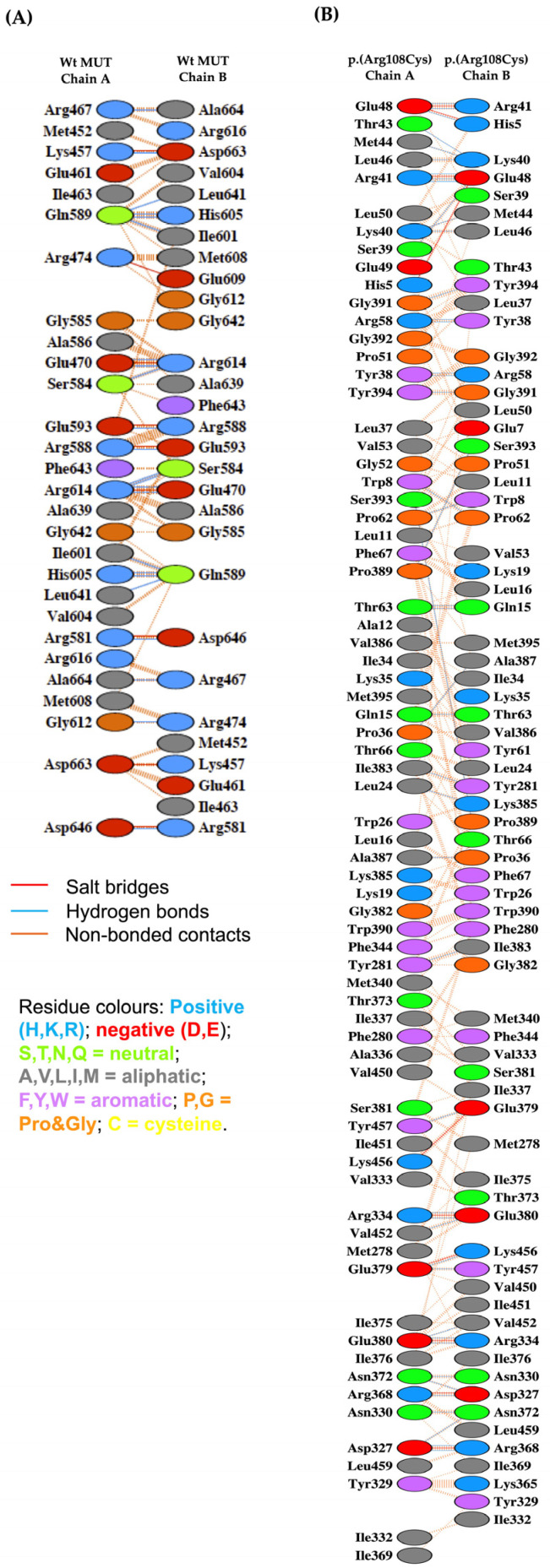
Interface amino acid interactions of the Wt MUT homodimer vs. NM_000255.4:c.322C>T or p.(Arg108Cys) homodimer. (**A**) Amino acids that constitute the interface between two Wt MUT monomers. (**B**) Amino acids that constitute the interface between two NM_000255.4:c.322C>T or p.(Arg108Cys) variant monomers.

We investigated whether the NM_000255.4:c.322C>T or p.(Arg108Cys) variant could induce changes in the dimerization interface of the homodimer. Thus, the structures resulting from MDS were subjected to protein–protein molecular docking assays. In this regard, the NM_000255.4:c.322C>T or p.(Arg108Cys)-NM_000255.4:c.322C>T or p.(Arg108Cys) dimer was performed. The best-ranked docking model (docking score = −1508.79) was used to analyze the amino acid residues of the interface. The resulting interface residues of such a model were compared with those of the Wt-Wt dimer determined from experimentally obtained atomic coordinates (PDB code: 2XIQ). Interestingly, the NM_000255.4:c.322C>T or p.(Arg108Cys) variant induced significant changes. For instance, in the Wt protein dimer, each monomer contributed with 1343 Å^2^ and 1346 Å^2^ of surface area involving 28 and 29 amino acids, respectively, and forming 23 hydrogen bonds. In contrast, in the NM_000255.4:c.322C>T or p.(Arg108Cys) homodimer, each monomer contributed with more than 2-fold the surface area of the Wt dimer, with 3386 Å^2^ and 3459 Å^2^ and involving 71 and 70 amino acids, with 46 hydrogen bonds formed. Moreover, it was found that the dimer formation in the NM_000255.4:c.322C>T or p.(Arg108Cys) variant did not align in the correct region of the protein, suggesting alterations in protein functionality. Figure 5 provides a detailed depiction of these interactions.

### 2.4. Changes in Secondary Structure Between Wt MUT and p.(Arg108Cys) Variant

As mentioned above, MDS analysis was carried out to predict the stability of both the wild-type protein and its variant p.(Arg108Cys) for comparison. Then, a more detailed comparison of the changes in the secondary structure between the Wt MUT and the NM_000255.4:c.322C>T or p.(Arg108Cys) variant is provided in Table 1. The structural analysis identified 51 changes in secondary structure motifs (17 strands and 34 helices), with the catalytic domain being the most affected. The number of strands in the Wt MUT decreased from 65 to 53 in the NM_000255.4:c.322C>T or p.(Arg108Cys) variant. Also, the number of helices constituted from 3 to 10 amino acid residues and decreased from 26 to 17 in the NM_000255.4:c.322C>T or p.(Arg108Cys) variant compared with the Wt MUT. The most dramatic changes observed were the complete loss of some helices in the NM_000255.4:c.322C>T or p.(Arg108Cys) variant.

### 2.5. Local Changes in MUT Exerted by Cys Substitution at Position 108

**Figure 6:**
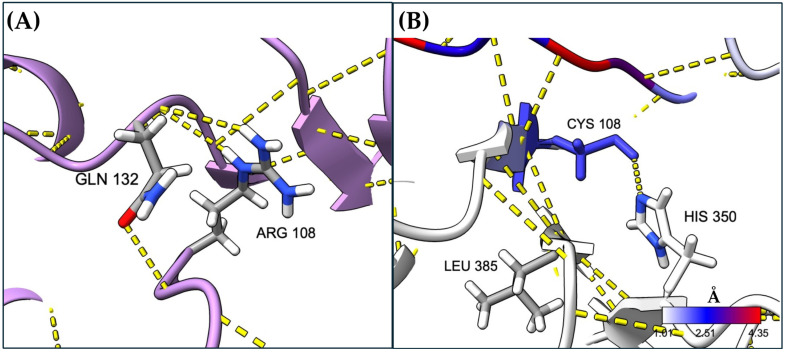
Local changes exerted by the arginine 108 residue: (**A**) change in the Wt MUT monomer by cysteine; (**B**) hydrogen bonds are shown with dotted yellow lines. Structures are visualized in ribbons and sticks.

The local changes surrounding the mutation site were analyzed, focusing on hydrogen bonds and other important parameters. We found that the mutation disrupted key interactions, such as hydrogen bonds, i.e., the Arg 108 residue usually forms bonds with Gln132, while Cys at position 108 interacts with His350 and Leu385 (Figure 6).

**Figure 7:**
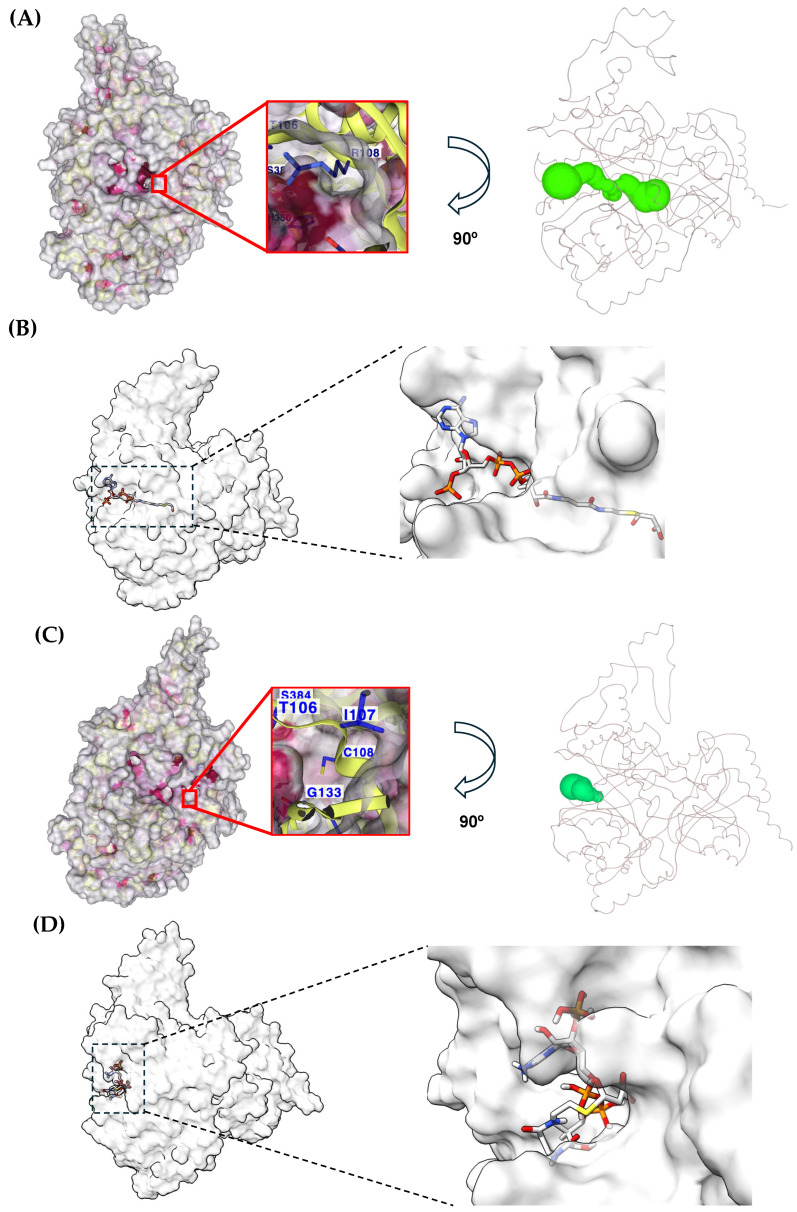
Differences in the cavity volume between Wt MUT and NM_000255.4:c.322C>T or p.(Arg108Cys) variant. The volume of the cavity of Wt MUT (**A**) is the smallest and shows the deepest tunnel (green-colored, right side), where the substrate is adequately bound. (**B**) The cavity of the p.(Arg108Cys) variant shows the largest volume (**C**) with a shortened tunnel (green-colored, right side), causing an aberrant accommodation of the substrate (**D**). The close-ups highlight the depth of the pockets and the disposition of the residue 108. MDS frames from the last 105–110 ns were used for the cavity analysis.

Also, the SASA significantly changed at position 108, whereas the SASA at position 108 in the Wt MUT was 48.8 Å^2^, and it was 2.8 Å^2^ in the NM_000255.4:c.322C>T or p.(Arg108Cys) variant. The cavity of the catalytic domain of Wt MUT and the NM_000255.4:c.322C>T or p.(Arg108Cys) variant were obtained and compared. We found that the volume of the Wt MUT cavity was 968 Å^3^ and shows a deep tunnel within the protein structure (Figure 7A). Such a topology permits the substrate to dock into the tunnel as previously described for the experimentally obtained X-ray structure (Figure 7B). On the other hand, the volume of the cavity of the NM_000255.4:c.322C>T or p.(Arg108Cys) variant increased to 1585 Å^3^. However, the tunnel topology of this region was shortened (Figure 7C) with a concomitant aberrant accommodation of the substrate (Figure 7D). In a close-up of the catalytic cavity, it can be observed that the Arg108 residue (Figure 7A) is exposed to the solvent within the cavity. In contrast, the Cys108 residue is buried in the cavity and weakly exposed to the solvent (Figure 7C).

## 3. Discussion

The main finding of this study is that the NM_000255.4:c.322C>T or p.(Arg108Cys) variant potentially yields such ostentatious topological changes in the secondary structure of the protein that can affect the oligomerization, as well as the three-dimensional arrangement of the catalytic pocket. These findings support a feasible explanation of the severity and high mortality (83%) of the phenotype observed in our homozygous patients. The clinical picture was characterized by gastrointestinal and neurological manifestations, recurrent infections, and dehydration, accompanied by metabolic acidosis, hyperammonemia, and anemia (Figure 1A,B). This phenotype is like the one described by other authors for other severe MMUT variants [19,20]. Moreover, the high mortality observed could be related to the late diagnosis and establishment of treatment in all the cases; thus, detecting in an early stage is essential to attenuate the severity and a fatal outcome.

The NM_000255.4:c.322C>T or p.(Arg108Cys) variant was first reported by Worgan et al. in a series of Hispanic patients with MMA living in Canada [17]. It has also been found in patients from other populations, such as the Saudi [21] and Chinese [20,22,23], and carriers have also been detected in the Indian population [24] https://databases.lovd.nl/shared/genes consulted on 13 January 2025. Interestingly, the geographic distribution of this variant in the present study is distributed in the central region of the country, known as “El Bajío”, which includes Querétaro, Guanajuato, and part of Zacatecas States (Figure 1C). Such a high prevalence of some genetic disorders in this geographical region has been documented, probably due to founder effects and endogamy practices [25,26].

Deepening the explanation of the severity of the NM_000255.4:c.322C>T or p.(Arg108Cys) variant studied herein, the MDS analysis revealed that the single Arg substitution for Cys at position 108 exerts significant structural effects both locally and at a long-range distance (Figure 1). At the local level, we found that the interaction between arginine 108 and glutamine 132 was lost when it was substituted by cysteine (Figure 6). This could be explained by the distance between the residues Cys 108 and Gln 132, which is long enough to avoid their interaction (Figure S1). This corroborates our previous prediction. When we performed in silico mutagenesis to generate the NM_000255.4:c.322C>T or p.(Arg108Cys) variant, we found that substituting arginine with a cysteine residue could induce the loss of key interactions that the arginine residue has with its neighboring amino acids and the substrate [16].

Furthermore, our study through MDS analysis supports that the NM_000255.4:c.322C>T or p.(Arg108Cys) variant induces significant topology changes in the secondary structure of the protein (Table 1). This occurs in all the domains but is more notable in the catalytic one. In this domain, we documented the most striking changes with the complete loss of three β-sheet helices and one gain. Moreover, several changes in the type of β-sheet occurred, i.e., the generation of crossover β-sheet connections instead of the beta bulge. It is known that beta bulges give flexibility to the sheet; thus, the substitution of beta bulge by crossover connections impairs the flexibility of that region [18]. In the linker domain, the type of β-sheet remains the same; however, the number of amino acid residues constituting each one changes slightly. Also, in the AdoCbl-binding domain, the topological distortions were subtle. However, some changes in the type of β-sheet occurred, mainly crossover changes, which were substituted by extended sheets.

As our MDS and docking studies show, the changes observed were beyond the secondary structure since the three-dimensional arrangement of the catalytic pocket was also altered (Figure 7). According to the crystallographic coordinates provided by Froese et al., the catalytic pocket shows a tunnel conformation, where the substrate gets in close contact with the amino acid residues Tyr110, His265, and Arg228 [27]. However, our docking analyses with the NM_000255.4:c.322C>T or p.(Arg108Cys) variant revealed that this pocket exhibited a shortened conformation, and the entry region got broader. Consequently, when docking the substrate against the NM_000255.4:c.322C>T or p.(Arg108Cys) variant, the substrate molecule was possessed in an aberrant conformation compared with its canonical accommodation in the Wt enzyme [5,27].

Our exploration of the oligomerization of the NM_000255.4:c.322C>T or p.(Arg108Cys) variant through docking showed a significant structural impairment since a notable increase in interactions was predicted. Moreover, the amino acid residues that constituted the Wt interface were utterly different in the NM_000255.4:c.322C>T or p.(Arg108Cys) variant (Figure 5). Mascarenhas et al. obtained the crystallographic structure of the MUT enzyme in complex with the substrate, the cofactor, and the MMAA chaperone. They found that the conformational arrangement of MUT suffers spatial modifications, i.e., a 180° rotation of the AdoCbl-binding domain is held, and that this movement is crucial for the catalytic mechanism [5]. Thus, if the amino acid residues that establish the interface change, then the formation of the enzymatic complex and the rotation needed during catalysis can be compromised.

The sum of all the above-mentioned changes could be extrapolated to the impaired protein functionality, as Chu et al. observed in a previous study. They performed the assay of propionate incorporation in fibroblasts from three homozygous patients for the NM_000255.4:c.322C>T or p.(Arg108Cys) variant. This analysis, which is an indirect way to measure the functionality of the MUT enzyme, revealed that compared to the standard control of propionate incorporation (11 ± 4 nmol/mg/18 h), in the fibroblasts from the patients, it decreased to a mean of 1.03 nmol/mg/18 h [28]. However, functional studies such as the protein’s enzymatic activity in patients’ fibroblasts are essential to elucidate the direct impact that this variant has on MUT functionality.

Despite some difficulties, MDS is nowadays capable of analyzing and predicting real-world processes, especially those that are poorly accessible or not accessible at all, through experiments [29]. In the present study, the molecular dynamics simulations of the NM_000255.4:c.322C>T or p.(Arg108Cys) MUT variant yielded valuable structural insights that allowed us to only predict the possible effect of the variant in the structure of the protein. Thus, further experimental studies are needed to corroborate the implications of the variant at the structural level, followed by research on molecules that could improve its structure and function.

## 4. Materials and Methods

### 4.1. Families and Patients

A descriptive, retrospective analysis of the medical files of six unrelated *MMUT* homozygous patients whose genotype is c.[322C];[322C>T] or p.[Arg108Cys];[Arg108Cys] was performed. The patients came from a cohort of families at the Laboratory of Inborn Errors of Metabolism of the National Institute of Pediatrics. The clinical, biochemical, and demographic data were collected from the patients’ files at diagnosis and at the outcome.

### 4.2. In Silico Analyses

In this study, we analyzed the structural changes caused by the NM_000255.4:c.322C>T or p.(Arg108Cys) variant in MUT using computational tools such as molecular dynamics and docking. To that end, the crystallographic atomic coordinates of the wild-type (Wt) MUT (Protein Data Bank (PDB) code: 2XIQ, https://www.rcsb.org/ accessed on 10 December 2024) were used for all the in-silico analyses [27]. The NM_000255.4:c.322C>T or p.(Arg108Cys) variant was obtained by mutagenesis in silico using the PyMOL software (PyMOL Molecular Graphics System, version 2.0 Schrödinger, LLC, New York, NY, USA [30]) based on the 2XIQ crystal of the Wt MUT. The structure of malonyl-CoA, a substrate analog, was also removed from the 2XIQ crystal. For the analyses, we started from the crystallographic structure of the Wt MUT and performed in silico mutagenesis to obtain the variant p.(Arg108Cys), as mentioned above. Both crystallographic structures were analyzed using MD, and comparisons were made between the MD of the Wt MUT and the MD of the NM_000255.4:c.322C>T or p.(Arg108Cys) variant.

### 4.3. Molecular Dynamics Simulations

The molecular dynamics simulations (MDSs) were performed in triplicate. The structures of the Wt MUT and the in silico constructed NM_000255.4:c.322C>T or p.(Arg108Cys) variant were used as starting configurations for MDS using the GROMACS software (version 2018.4, Stockholm, Sweden) with 110 nanoseconds (ns) final simulation at a temperature of 300 K. The system was solvated by adding water molecules in a dodecahedron with a minimum distance from the wall of 10 Å using the TIP3P water model. Afterward, the system was neutralized by adding Na^+^/Cl^−^ ions and energy-minimized by the steepest descent algorithm (50,000 times). The equilibrium steps were conducted at 300 K in two steps: (1) the ligand was simulated at NVT conditions (constant number of particles, volume, and temperature) using a V-rescale thermostat considering a time constant (tau_t) of 0.1 ps obtaining velocities according to a Maxwell–Boltzmann distribution; and (2) the ligand was simulated at NPT conditions (constant number of particles, pressure at 1 atm, and temperature) utilizing a V-rescale thermostat and a Berendsen barostat with time constants (tau_t and tau_p) of 0.1 and 2.0 ps, respectively. Each step was achieved at 100 ps. The stability of the complex was determined by root mean square deviation (RMSD) and root mean square fluctuation (RMSF) calculations using the GROMACS software tools [31,32,33]. To evaluate the structural deviations between the Wt MUT enzyme and the one with the NM_000255.4:c.322C>T or p.(Arg108Cys) variant, we compared the RMSD of the main chain alpha carbons of both enzymes during MD simulations. Also, the structures extracted from the MD simulations (frames from 105–110 ns) were used to compare the long-range changes based on RMSD calculation.

After the MDS analysis, we aimed to investigate whether the NM_000255.4:c.322C>T or p.(Arg108Cys) variant could provoke changes in the cavity of the catalytic domain. To that end, the detection and analysis of cavities in the Wt MUT and the NM_000255.4:c.322C>T or p.(Arg108Cys) variant were determined with the CavityPlus tool [34]. The web-based tool MOLEonline was also used to analyze the catalytic site channel (https://mole.upol.cz/ accessed on 16 December 2024 [35]).

We also wondered whether the NM_000255.4:c.322C>T or p.(Arg108Cys) variant could cause changes in the amino acid residues that participate in the dimeric interface. Thus, we performed molecular docking between two monomers of the NM_000255.4:c.322C>T or p.(Arg108Cys) variant and compared the interface interactions with the previously established canonical interactions of the Wt-Wt homodimer. For molecular docking, the HDOCK server (http://hdock.phys.hust.edu.cn/ accessed on 16 January 2025 [36]) was used after the energy minimization of the structures with the Yasara minimization server (https://www.yasara.org/minimizationserver.htm accessed on 6 January 2025 [37]).

Afterwards, the interface interactions between the MUT monomers of the Wt enzyme and the NM_000255.4:c.322C>T or p.(Arg108Cys) variant were evaluated using the PDBsum web server of the European Bioinformatics Institute (https://www.ebi.ac.uk/thornton-srv/databases/pdbsum/ accessed on 20 January 2025 [38]).

### 4.4. Analysis of Simulated Trajectories

Following the production MDS run for 110 ns, the resultant trajectory files were analyzed using Gromacs utilities such as “gmx rms”, “gmx rmsf”, “gmx gyrate”, and “gmx sasa” for RMSD, RMSF, Rgyr, and SASA, respectively. The data were examined for a minimum steady trajectory of 60 ns in a window size of 15 ns. Chimerax software (version 1.9, San Francisco, CA, USA) was used to visualize the structural figures [39]. Xmgrace [40] tool (version 2, Portland, OR, USA) was used to generate the graphs and plots. These data analyses were performed to check for the rigidity, compactness, fluctuations, and stability of the Wt MUT and p.(Arg108Cys) variant.

### 4.5. Molecular Docking

Protein–protein molecular docking was performed to predict the molecular interactions between the monomer of the p.(Arg108Cys) variant after the MDS run for 110 ns with the same monomer p.(Arg108Cys) to elicit the potential dimer that could be formed in the homozygous individuals. These docking simulations were performed using the HDOCK server (http://hdock.phys.hust.edu.cn/ accessed on 22 January 2025) [41]. The PDBsum web server of the European Bioinformatics Institute [38] was used to analyze the interface interactions of the p.(Arg108Cys)/p.(Arg108Cys) dimer, and the amino acid residues of the interface were compared with those of the Wt MUT/Wt MUT dimer from the crystallographic atomic coordinates from the structure with PDB code 2XIQ. To predict the molecular interactions between the substrate (methylmalonyl-CoA) and MDS structures of Wt MUT and p.(Arg108Cys), targeted molecular dockings were performed by using AutoDock Vina (version 1.2.0 https://vina.scripps.edu/ accessed on 29 January 2025) [42]. To that end, the search space along the catalytic region was defined with a box of 23 Å × 22 Å × 22 Å in size.

### 4.6. Statistical Analyses

Descriptive statistics for the clinical data were performed. The normal distribution of the data was determined with the Shapiro test. One-way ANOVA followed by Dunn’s multiple comparisons adjustment was used to analyze the normally distributed data. Non-parametric distributed data were analyzed using the Mann–Whitney U-test and Dunn’s multiple comparisons adjustment test. The results are expressed as mean ± SD or median and interquartile range (IQR) for data with parametric and non-parametric distributions, respectively. A *p*-value of <0.05 was considered statistically significant (* *p* < 0.05; ** *p* < 0.01; *** *p* < 0.001; **** *p* < 0.0001). For these analyses, GraphPad Prism (version 10.4.1, Boston, MA, USA) was used.

## 5. Conclusions

Our findings strongly support that the substitution of Arg for Cys severely impacts the structure of MUT. It is worth noting that such a local single amino acid substitution exerts long-range distance changes that potentially contribute to the severe phenotype observed in homozygous patients. Ex vivo functional studies could confirm these predictions, such as enzymatic activity measurements in the fibroblasts of patients.

## Figures and Tables

**Table 1:** Comparison of changes in the structural motifs * of the wild-type and NM_000255.4:c.322C>T or p.(Arg108Cys) variant of methylmalonyl-CoA mutase by protein domain after molecular dynamics.

		Wt				Arg108Cys			
	Structural Motif	Start	End	Type of b-Sheet	Number of Residues	Start	End	Type of Sheet	Number of Residues
**Catalytic domain**	Strand	Thr106	Ala111	B	6	Thr106	Cys108	B	3
						Tyr110	Ala11	C	2
	Helix	Val116	Lys128	H	13	Val116	Ile127	H	12
	Strand	Gly133	Val136	B	4	Ser135	Val136	C	2
	Helix	Pro151	Val153	G	3	Lost			
	Strand	Val184	Met186	B	3	Ser185	Met186	C	2
	Helix	Lys210	Lys212	G	3	Lost			
	Strand	Gly215	Ile217	B	3	Gly215	Thr216	D	2
	Helix	Leu222	Met226	H	5	Leu22	Phe225	G	4
	Strand	Ile259	Ser262	B	4	Ile259	Ile261	B	3
	Helix	Ala302	Arg304	G	3	Lost			
		Phe315	Phe337	H	23	Phe315	Met336	H	22
	Strand	Ala349	Thr353	B	5	Ala349	Gln352	B	4
	Helix	Asn365	Phe379	H	15	Pro363	Asn365	G	3
						Ile367	Phe379	H	13
		Absent				Glu392	Leu394	G	3
**Linker domain**	Helix	Met455	Glu461	H	7	Gly454	Glu461	H	8
		Asn507	Ser524	H	18	Asn507	Lys522	H	16
		Ile548	Arg557	H	10	Leu549	Ala558	H	10
		Ala586	Phe591	H	6	Tyr587	Phe591	H	5
**AdoCbl-Binding Domain**	Strand	Ala668	Thr673	C	6	Ala668	Val671	E	4
	Helix	His678	Ser691	H	14	Val682	Ser691	H	10
	Strand	Leu698	Gly703	C	6	Leu698	Gly702	E	5
		Asn720	Phe722	C	3	Val721	Phe722	E	2

* According to the topology connections nomenclature of Richardson [18]. Abbreviations: B, beta bulge; C, crossover; D, direct; E, extended; G, gamma turn; H, Hairpin.

## Data Availability

Data can be available at a reasonable request.

## Supplementary Materials

The supporting information can be downloaded at: https://www.mdpi.com/article/10.3390/ijms26072887/s1.

## Abbreviations

The following abbreviations are used in this manuscript:

AdoCbl	adenosylcobalamin
C3	Propionylcarnitine
MDS	molecular dynamics simulations
MMA	methylmalonic acidemia
MMAA	metabolism of cobalamin-associated A
MMAB	metabolism of cobalamin-associated B
MUT	methylmalonyl-CoA mutase
*MMUT*	methylmalonyl-CoA mutase gene
NPT	constant number of particles, pressure at 1 ATM, and temperature
NVT	constant number of particles, volume, and temperature
OH-2 Cbl	hydroxocobalamin
PDB	Protein Data Bank
RMSD	root mean square deviation
RMSF	root mean square fluctuation
Wt	wild-type
