# Supplementary material for: The Arg108Cys Variant of Methylmalonyl-CoA Mutase: Clinical Implications for the Mexican Population Based on Molecular Dynamics and Docking
Source: Int J Mol Sci. 2025 Mar 22;26(7):2887. doi: 10.3390/ijms26072887 (PMC11988910; doi:10.3390/ijms26072887)
Supplement: Supplementary file 1 [file ijms-26-02887-s001.zip › ijms-3501172-supplementary.pdf]

International Journal of
Molecular Sciences

MDPI

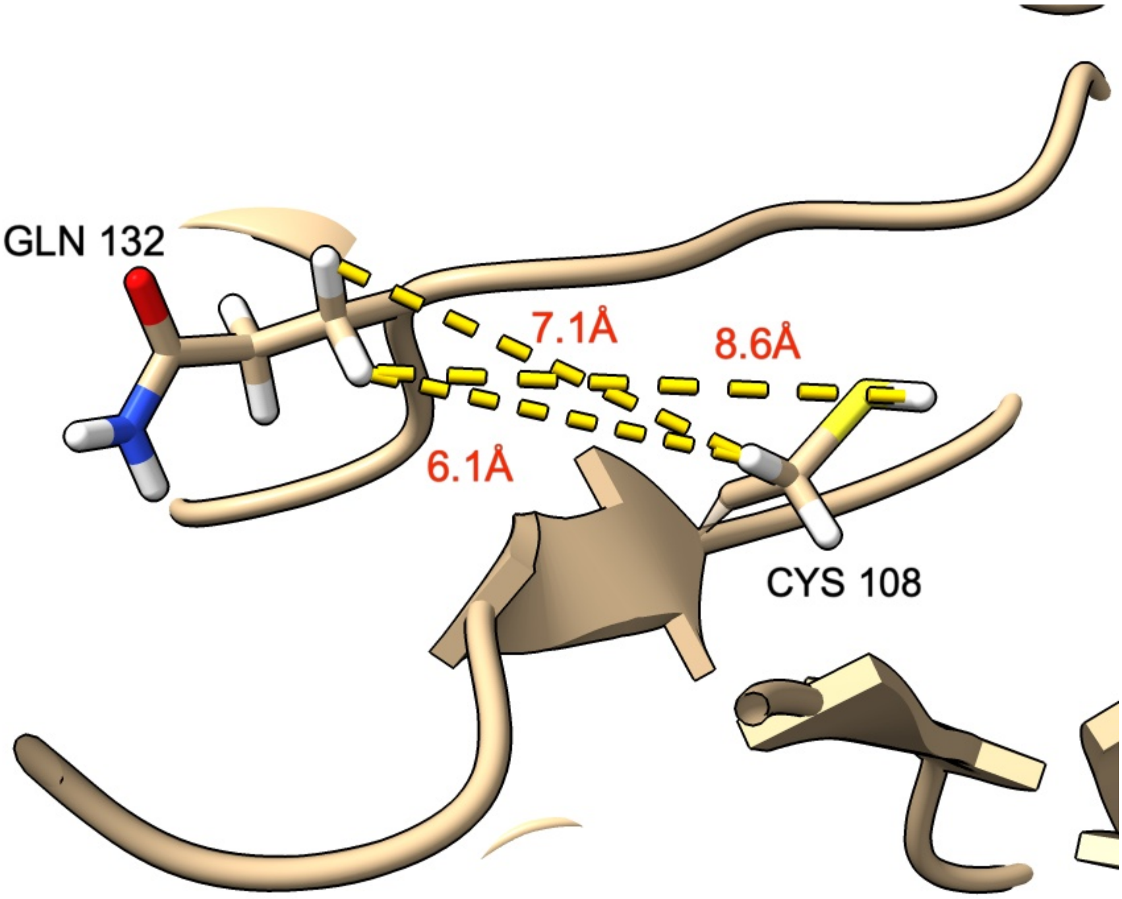

Figure S1. Distance between glutamine 132 residue and cysteine 108 residue in the p.Arg108C variant of methylmalonyl-CoA mutase.

 https://doi.org/10.3390/ijms26072887
